# Histone H2AX promotes metastatic progression by preserving glycolysis via hexokinase-2

**DOI:** 10.1038/s41598-022-07675-6

**Published:** 2022-03-08

**Authors:** Yue Liu, Haojian Li, Crystal N. Wilson, Hui Jen Bai, Myriem Boufraqech, Urbain Weyemi

**Affiliations:** 1 Department of Molecular Biosciences, The University of Texas at Austin, 2506 Speedway, Austin, TX 78712 USA; 2 Institute for Cellular and Molecular Biology, The University of Texas at Austin, Austin, TX 78712 USA

**Keywords:** Breast cancer, Cancer genomics, Cancer metabolism, Metastasis

## Abstract

Genomic stability is essential for organismal development, cellular homeostasis, and survival. The DNA double-strand breaks are particularly deleterious, creating an environment prone to cellular transformation and oncogenic activation. The histone variant H2AX is an essential component of the nucleosome responsible for initiating the early steps of the DNA repair process. H2AX maintains genomic stability by initiating a signaling cascade that collectively functions to promote DNA double-strand breaks repair. Recent advances have linked genomic stability to energetic metabolism, and alterations in metabolism were found to interfere with genome maintenance. Utilizing genome-wide transcripts profiling to identify differentially-expressed genes involved in energetic metabolism, we compared control and H2AX-deficient metastatic breast cancer cell lines, and found that H2AX loss leads to the repression of key genes regulating glycolysis, with a prominent effect on hexokinase-2 (HK2). These observations are substantiated by evidence that H2AX loss compromises glycolysis, effect which was reversed by ectopic expression of HK2. Utilizing models of experimental metastasis, we found that H2AX silencing halts progression of metastatic breast cancer cells MDA-MB-231. Most interestingly, ectopic expression of HK2 in H2AX-deficient cells restores their metastatic potential. Using multiple publicly available datasets, we found a significantly strong positive correlation between H2AX expression levels in patients with invasive breast cancer, and levels of glycolysis genes, particularly HK2. These observations are consistent with the evidence that high H2AX expression is associated with shorter distant metastasis-free survival. Our findings reveal a role for histone H2AX in controlling the metastatic ability of breast cancer cells via maintenance of HK2-driven glycolysis.

## Introduction

Most cancer deaths are due to metastatic disease^1^. Understanding how cells migrate from their primary site to seed new tumors throughout the body is a longstanding challenge in developing novel cancer treatments. Current models propose that invasive cancer cells undergo phenotypic changes involving epithelial to mesenchymal transition (EMT). EMT is characterized by the loss of cell polarity, cell–cell adhesion, and the acquisition of migratory and invasive properties^1^. Recently, we showed that deficiency in the DNA repair histone variant H2AX in colon cancer cells led to chromatin remodeling and activation of key transcription factors involved in the EMT in colon cancer cells, but with limited impact on the metastatic potential in immunocompromised mice^2^. Other findings implicated additional chromatin-based DNA repair proteins including BRG1, histone macroH2A.1 and BRCA1 in abnormal epithelial differentiation^3–5^.

There is mounting evidence on the requirement for enhanced energetic metabolism in invasive cancer cells to establish metastases in a nutrients-poor tumor microenvironment^6,7^, particularly in invasive breast cancer cells^6^. For instance, silencing of mitochondrial biogenesis through repression of the transcription co-activator PGC-1α is accompanied by significant mitigation of the invasive ability of breast cancer cells, limiting their potential to establish tumor nodules at distant sites^6^. Thus, impairment of energetic metabolism in cancer cells reduces metastatic potential of highly invasive cancer cells. Normal mammalian cells maintain their energetic metabolism via multiple pathways coordinating the conversion of aerobic glucose into pyruvate. However, in cancer cells, there is an increase in the rate of glucose uptake and preferential production of lactate, even in normoxic conditions. This process is known as the Warburg effect^8,9^. The Warburg effect provides metabolites for cancer cell proliferation and promotes resistance to apoptosis. Our prior research revealed repression of mitochondrial energetic metabolic genes in untransformed H2AX-deficient cells^10^. We hypothesize that impaired energetic metabolism resulting from H2AX loss increases the ability of metastatic cancer cells to heavily rely on reduced metabolism, thereby limiting the energy available for their proliferation in a nutrients-poor tumor environment.

Utilizing transcriptomics to determine whether H2AX loss affects metabolic genes in highly invasive triple-negative breast cancer cells, we found that H2AX-deficient cells exhibit repression of glycolysis genes. Notably, H2AX loss leads to a major repression of genes coordinating glycolysis, particularly hexokinase-2 (HK2). HK2 is one of the major frontline enzymes required for glycolysis and cancer cell survival^11,12^. Recent findings demonstrated that HK2 elicits multiple key steps responsible for cancer progression and resistance to current therapies^13–16^. Overexpression of HK2 in H2AX-deficient cells partly rescues glycolysis as evidenced by restoration of lactate production in MDA-MB-231 cells. When injected into immunocompromised mice, H2AX-deficient cells exhibit reduced metastases in the lung, and failed to establish nodules at distant sites. Ectopic expression of HK2 in H2AX-depleted cells restores their metastatic potential. Our data suggest that H2AX-deficient cells failure to metastasize may reflect a glycolysis-dependent energetic metabolic dysfunction. Our study reveals unprecedented evidence of a role for histone H2AX in promoting metastatic ability of invasive breast cancer cells, potentially via transcriptional regulation of hexokinase-2 and glycolysis maintenance.

## Results and discussion

### H2AX depletion represses glycolytic genes in invasive breast cancer cells

**Figure 1:**
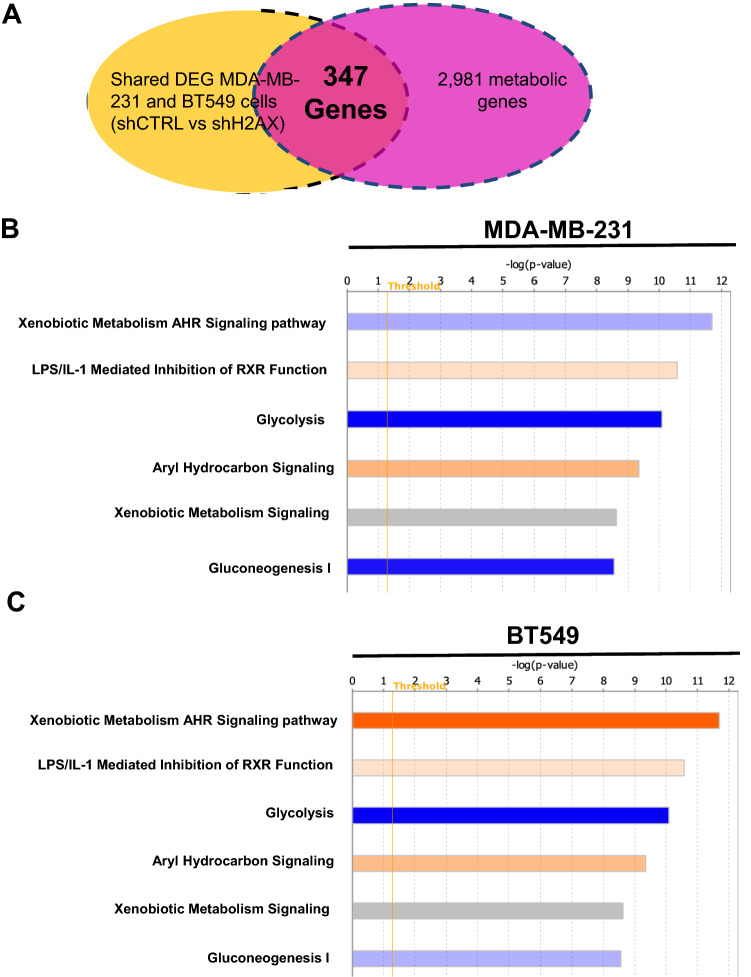
H2AX is a key player in the regulation of glycolysis in triple-negative breast cancer cells. (**A**) MDA-MB-231 and BT549 cells were transfected with scrambled short hairpin RNA (shCTRL) or with short hairpin RNA against *H2AFX* (shH2AX), and the resulting cells were used for transcriptomics to generate Differentially Expressed Genes (DEG) between shCTRL and shH2AX. DEG were merged with 2981 metabolic genes to identify genes regulated by H2AX. The output from the overlay between the two genes sets revealed 347 genes commonly shared and consistent in both MDA-MB-231 and BT549 cells. (**B**,**C**) Ingenuity Pathway Analysis (*IPA*) was used to establish cellular pathways in which the 347 metabolic genes are involved. The output revealed enrichment for multiple pathways involved in metabolic reprogramming of cancer cells. Glycolysis pathway is significantly repressed in both cell lines. Blue bars represent repressed pathways, and orange bars represent activated pathways. Grey bars indicate a pathway with fewer molecules and limited enrichment for a prediction. The height of the bar represents the p-value. The yellow line indicates the log (p-value) threshold of significance (1.3), which corresponds to p-value of 0.05. Statistical significance was determined by Fishers Test.

We previously demonstrated that histone H2AX controls metabolic pathways involving oxidative phosphorylation and mitochondrial homeostasis^17–19^. Mounting evidence point to a role for energetic metabolic pathways such as glycolysis, and oxidative phosphorylation in promoting metastatic cancer cells’ ability to establish tumors at distant sites^6,8,9^. Likewise, genomic instability coupled with energetic metabolism fuel cancer cells to survive in the nutrient-poor tumor microenvironment. To determine the role of histone H2AX in energetic metabolism, we performed a genome-wide differential gene expression (DEG) analysis comparing control triple-negative MDA-MB-231 and BT549 breast cancer cells, with H2AX-deficient cells generated using short hairpin RNA interference approach. The output from the DEG analysis yields 3956 genes in MDA-MB-231 cells and 5377 genes in BT549 cells. These DEG between control cells and H2AX-deficient cells were merged with 2981 metabolic genes^20^. The output from the overlay yields 347 genes prominently involved in metabolic pathways and dysregulated in cells deficient for H2AX (Fig. 1A). Pathway analysis on these 347 metabolic genes revealed a significant enrichment for multiple signaling pathways involved in cancer, with the glycolysis pathway ranking as the most repressed across cell lines (Fig. 1B,C).

**Figure 2:**
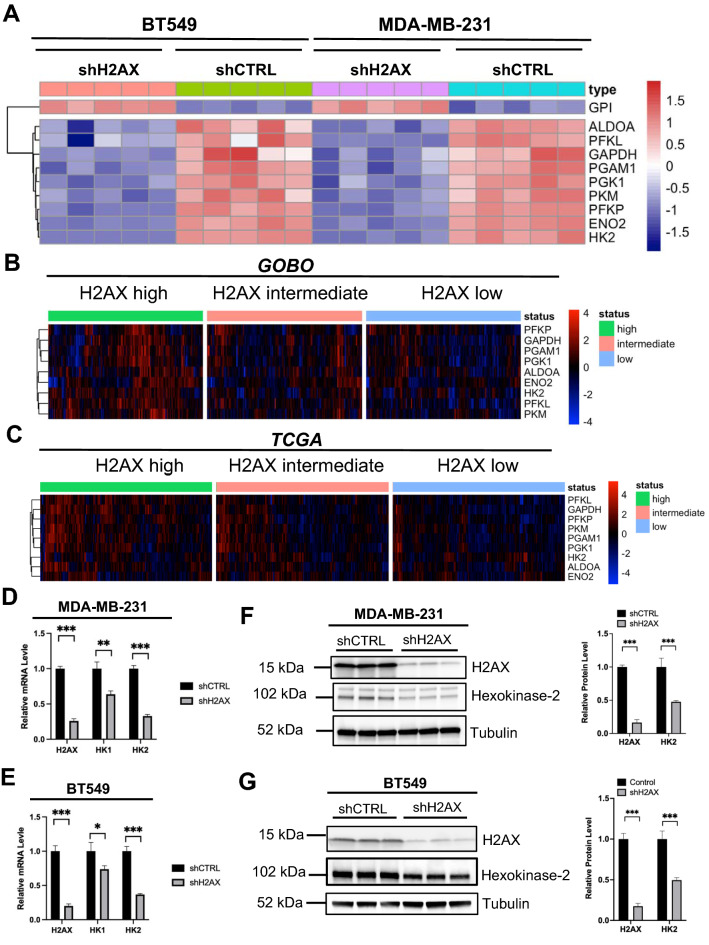
Down-regulation of H2AX promotes repression of key glycolysis genes. (**A**) Heatmap of the the ten genes found to be enriched in the glycolysis signaling pathway in *IPA*. Note that nine out of the ten genes were significantly repressed in cells deficient for H2AX, with only Glucose-6-Phosphate Isomerase (GPI) activated upon H2AX silencing. The numbering refers to independent replicates for either shCTRL sample or shH2AX sample. (**B**) The nine glycolysis genes repressed in H2AX-deficient cells were analyzed for their expression level in the *gene expression-based outcome for breast cancer online (GOBO)* dataset and compared to that of H2AX in the same patients. H2AX expression is significantly correlated with most glycolysis genes across specimens. (**C**) Similar comparison was performed in human invasive breast cancer samples available in the *Cancer Genome Atlas (TCGA)* dataset. (**D**,**E**) MDA-MB-231 cells (**D**) and BT549 cells (**E**) were transfected with either scrambled short hairpin RNA (shCTRL) or with short hairpin RNA against *H2AFX* (shH2AX), and transcript levels of HK1 and HK2 was analyzed using real-time PCR. Expression values are relative fold change for gene transcripts normalized to Actin transcript (Gene/Actin ratio). Error bars represent S.E.M. (*n* = 3). Statistical significance was determined by a two-tailed, unpaired Student’s *t*-test. (**F**,**G**). MDA-MB-231 cells (**F**) and BT549 cells (**G**) were transfected with either scrambled short hairpin RNA (shCTRL) or with short hairpin RNA against *H2AFX* (shH2AX) and cells were used for immunoblot analysis of H2AX and HK2 with tubulin detected as loading control. Statistical significance was determined by a two-tailed, unpaired Student’s *t*-test. *pvalue < 0.05, **p value ≤ 0.001, ***p value ≤ 0.0001, ns: non-significant.

Further analysis of genes found to be enriched in glycolysis pathway revealed consistent repression of nine essential glycolysis genes across cell lines including hexokinase-2 (*HK2*), Aldose, Fructose-Biphosphate A (*ALDOA*), Pyruvate Kinase M1/2 (*PKM*), and phosphoglycerate Mutase 1 (*PGAM1*) (Fig. 2A). *HK2* encodes for hexokinase-2, a rate-limiting enzyme for glycolysis. These findings imply a strong correlation between H2AX expression and genes essential for glycolysis. To further confirm this novel link between H2AX and glycolysis, we conducted a correlation analysis comparing H2AX expression level with the nine glycolysis genes obtained from the transcriptomics output in human invasive breast cancer tissues samples available in both the *Cancer Genome Atlas (TCGA)* and the *gene expression-based outcome for breast cancer online (GOBO)* datasets^21^. Glycolysis genes were clustered based on H2AX transcript levels evenly separated into 3 groups, and the output was used to generate a heatmap. The findings indicate that the transcript level of H2AX is significantly correlated with glycolysis genes across all specimens in *GOBO*, while the correlation is found non significant for HK2 in *TCGA* presumably owing to the fewer number of metastatic specimens in this cohort. Specifically, patients with high H2AX exhibit high levels of glycolysis genes, while patients with lower H2AX have significantly repressed expression of the same genes (Fig. 2B,C). The transcriptional repression of HK2 in H2AX-deficient cells was confirmed using real-time quantitative PCR (RT-qPCR) (Fig. 2D,E). Histone H2AX is primarily described for its role in DNA repair and for the recruitment of repair partners proteins to the DNA-damaged sites. However, the observations that H2AX appeared to significantly influence transcript level of HK2 suggest a role for H2AX in transcriptionally regulating HK2 expression. Indeed, H2AX loss resulted in a 25% decrease in HK2 promoter activity. By contrast, the promoter activity of *ACTB* (beta-actin), a gene known not to be involved in glycolysis, remains relatively unchanged (Supplementary Fig. 1). Western blot analysis showed an over 40% decrease in HK2 protein levels in H2AX-deficient cells in both cell lines (Fig. 2F,G). Taken together, these findings reveal a previously unknown interplay between histone H2AX and glycolysis genes. Particularly, the repression of HK2 following H2AX silencing suggests a potential for H2AX to control genes involved in the early steps of glycolysis. These findings are in line with previous reports demonstrating the importance of histone H2AX in mediating cancer cells survival^22^. In fact, the findings established that activation of histone H2AX through its phosphorylation in response to DNA damage triggers HIF-1α enrichment in the nucleus of invasive breast cancer cells, with a subsequent overactivation of HIF-1α signaling. This activation is selectively associated with an accelerated cancer progression and metastasis. Indeed, H2AX depletion resulted in a significant decrease in cell proliferation associated with a higher level of apoptosis (Supplementary Fig. 2A,B). Unbiased analysis comparing differential pathways enrichment in both control and H2AX-deficient cells revealed that genes controlling activation of apoptosis are significantly overexpressed in H2AX-deficient cells (Supplementary Fig. 2C–E). Taken together, our new findings reveal a unique link between H2AX and glycolysis, pointing to a potential for H2AX to control energetic metabolism in invasive breast cancer cells’ survival.

### Ectopic expression of hexokinase-2 restores glycolysis in H2AX-deficient cells

**Figure 3:**
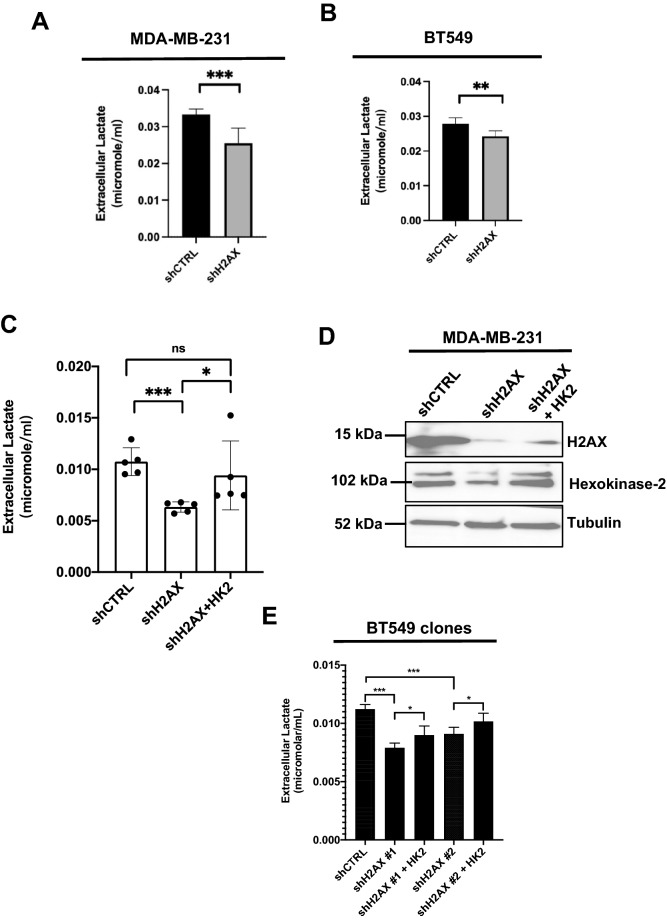
H2AX silencing leads to reduced glycolysis in invasive breast cancer cells. (**A**,**B**) H2AX silencing resulted in a 20% diminution in glycolysis level in both MDA-MB-231 (**A**) and BT549 cells (**B**). Level of glycolysis was detected by measurement of extracellular lactate production as described in “Methods” section. Error bars represent S.E.M. (*n* = 9). (**C**) Extracellular lactate level in MDA-MB-231 control cells (shCTRL), H2AX-deficient cells (shH2AX), and H2AX-deficient cells in which HK2 expression was reintroduced (shH2AX + HK2). (**D**) Cells described in (**C**) were subjected to immunoblot for the detection of protein levels for H2AX, HK2, and tubulin. (**E**) BT-549 cells were infected with either scrambled short hairpin RNA, or with short hairpin RNA against *H2AFX* (shH2AX), and cells were selected in the presence of puromycin to establish stable clones (shH2AX #1, and shH2AX #2). These control and H2AX-deficient cells were transfected with either control vector (shCTRL) or with vector expressing HK2 (shH2AX + HK2), and the resulting cells were used for quantification of extracellular lactate level. Statistical significance was determined by a two-tailed, unpaired Student’s *t*-test. Error bars represent S.E.M. (*n* = 6). *p value < 0.05, ***p value ≤ 0.0001, ns: non-significant.

Hexokinase-2 was re-expressed in H2AX-deficient cells to examine whether the metabolic characteristics of the parental MDA-MB-231 cells would be restored. H2AX silencing leads to a 20% decrease in glycolysis as evidenced by level of extracellular lactate production (Fig. 3A,B). Ectopic expression of HK2 restores lactate level (Fig. 3C,D), thereby implying that HK2 may mediate the role of H2AX in glycolysis. A similar pattern of improved glycolysis was found in two independent H2AX-silenced clones in which HK2 expression was restored (Fig. 3E). These findings suggest that HK2 mediates glycolytic metabolism induced by H2AX. While studies have demonstrated the role of HK2 in cancer progression and metastasis, the prominent contribution of H2AX in HK2-driven glycolysis is strikingly novel. These new findings establish a potential dialogue between histone H2AX and key glycolysis genes required for cancer cells survival.

### Histone H2AX loss abrogates metastatic progression of invasive breast cancer cells

**Figure 4:**
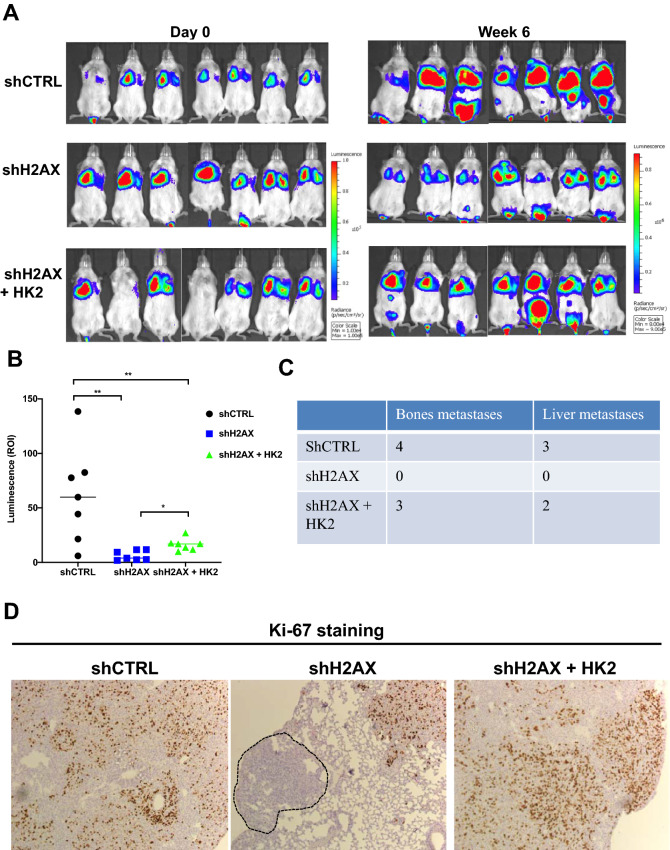
HK2 re-expression in H2AX-deficient cells partly restores metastatic colonization in the lung. (**A**) MDA-MB-231 control cells with empty vector (shCTRL), H2AX-deficient cells with empty vector (shH2AX), and H2AX-deficient in which HK2 was reintroduced (shH2AX + HK2) were inoculated via the tail veins of immunocompromised NOD SCID gamma mice, and animals were used for bioluminescence-based imaging from one week up to six weeks. The extent to which tumor cells exhibit growth and metastatic progression was monitored. Control cells (shCTRL) showed significantly elevated tumor growth in the lung as well as distant metastases to bones and liver, while H2AX-deficient cells showed a 60% reduction in tumor growth with no detectable distant metastases. Ectopic expression of HK2 resulted in a partial restoration of growth and metastastic potential. (**B**) Quantification of the luminescence detected in the lung. (**C**) The extent to which tumor cells metastasize outside the lung was analyzed by monitoring the presence of bioluminescence in the body. Note that luminescence was primarily detected in bones and liver specifically when animal were injected with either control cells (shCTRL) or with H2AX-deficient cells ectopically expessing HK2 (shH2AX + HK2). (**D**) Representative Ki-67-stained lung sections from mice injected with control cells with empty vector (shCTRL), H2AX-deficient cells with empty vector (shH2AX) and H2AX-deficient cells in which HK2 was reexpressed (shH2AX + HK2). Dashed lines indicate tumor regions in mice bearing H2AX-deficient cells with negative Ki-67 staining, implying presence of less aggressive tumor cells. Statistical significance was determined by a two-tailed, unpaired Student’s *t*-test. Error bars represent S.E.M. (*n* = 7 per group). *pvalue < 0.05; **p value ≤ 0.001.

Metastasis is the main cause of death in patients with breast cancer^23–25^. Among all forms of breast cancer, triple-negative breast cancer (TNBC) has the poorest outcome because of its high potential of metastatic progression, and the lack of effective targeted treatments^6,25,26^. The importance of metabolism in the progression of TNBC is gradually being investigated, but the dialogue between genomic instability and altered metabolism has been poorly studied. Insights into how metabolic reprogramming connects genomic instability to metastatic progression in TNBC is still unclear. Mounting evidence in the recent years have pointed to the notion that only a fraction of cancer cells detach from the primary tumor to migrate and invade into the bloodstream, and establish metastases at distant sites^16,27,28^. Reports have shown that the Warburg effect endows certain invasive cancer cells with the potential to evade excess lack of nutrients and stress to increase metastatic potential^29^. Compromising this pathway may repress cancer metastasis and cancer cells survival in the tumor microenvironment. To investigate how glycolysis impairment due to H2AX loss impacts the metastatic colonization potential of TNBC, we injected control cells, H2AX-deficient cells, and H2AX-deficient cells in which HK2 was ectopically expressed into the tail veins of immunocompromised mice and analyzed tumor growth in the lung, and metastatic progression to distant organs using bioluminescence imaging. H2AX silencing leads to a 60% reduction in tumor growth in the lung, while HK2 re-expression in H2AX-deficient cells partly restores tumor progression (Fig. 4A,B). These observations are particularly substantiated by the findings that mice injected with either parental cells or H2AX-deficient cells with ectopic HK2 expression exhibit higher number of bones and liver metastases (Fig. 4C). Imaging of lung metastasis 6 weeks post tumor inoculation confirms evidence of reduced metastatic potential in cells depleted for H2AX expression, while HK2 reactivation is found to foster the metastatic ability of cancer cells (Supplementary Fig. 3). When stained with Ki-67, a marker for highly proliferating cells, we found that H2AX-deficient tumor cells exhibit impaired proliferative ability in vivo as revealed by tumor sites lacking positive staining for Ki-67, while ectopic expression of HK2 in H2AX-deficient cells restore their highly aggressive proliferative behavior (Fig. 4D).

**Figure 5:**
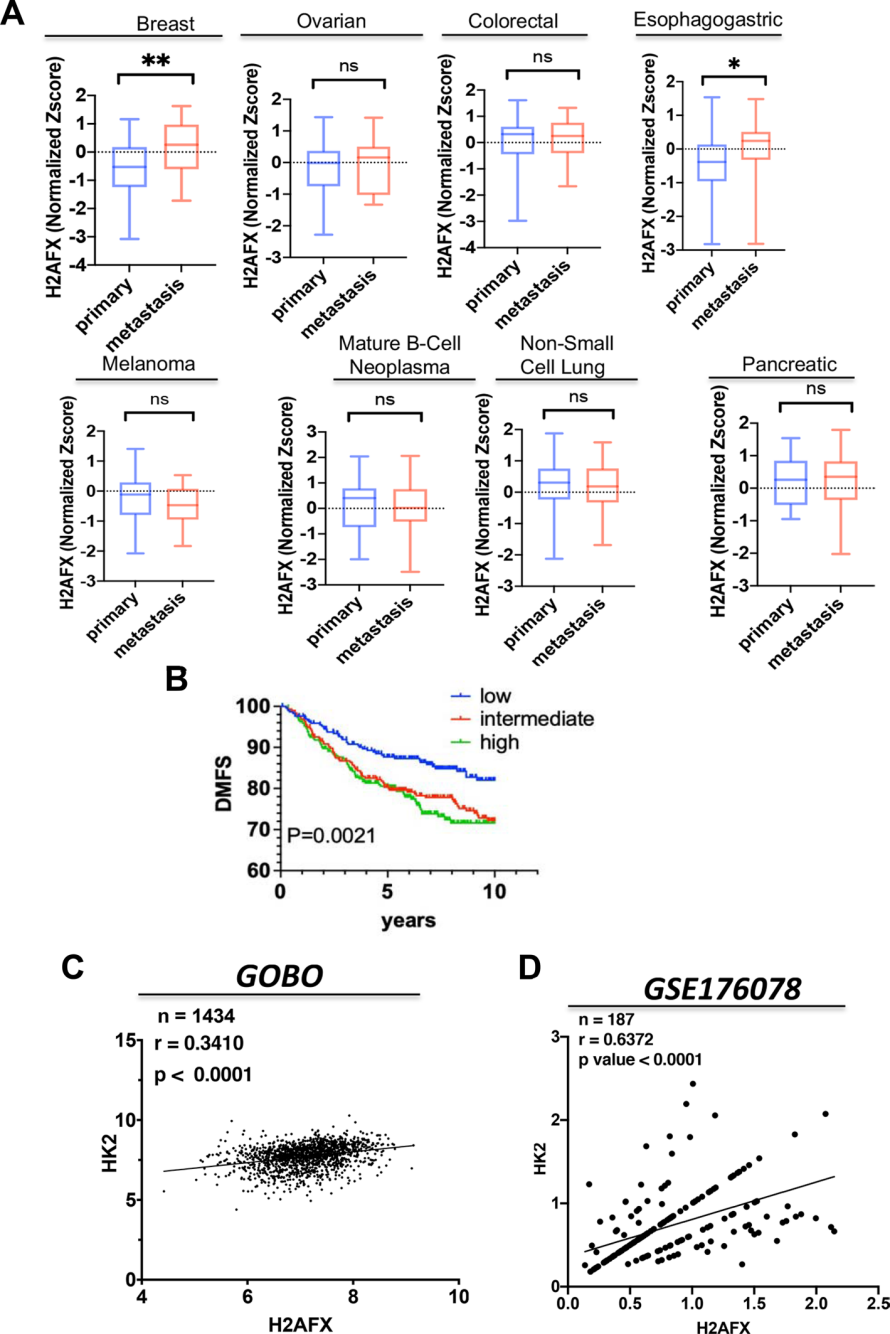
H2AX expression is correlated with that of HK2 and with survival rate in patients with invasive breast cancer. (**A**) Dataset from Cancer Cell line Encyclopedia (CCLE) was utilized to assess level of H2AX transcript comparing cells derived from primary sites with the ones from metastatic sites. CCLE normalized expression data and clinical information were downloaded from cBioportal. Breast (n = 29 primary *vs.* n = 26 metastasis), colorectal (n = 48 primary *vs.* n = 11 metastasis), esophagogastric (n = 66 primary *vs.* n = 27 metastasis), mature B-cell neoplasms (n = 39 primary *vs.* n = 15 metastasis), melanoma (n = 31 primary *vs.* n = 25 metastasis), non-small cell lung (n = 75 primary *vs.* n = 50 metastasis), ovarian (n = 34 primary *vs.* n = 15 metastasis), pancreatic (n = 26 primary *vs.* n = 15 metastasis), and small cell lung (n = 18 primary *vs.* n = 32 metastasis). For breast, **p value = 0.0092; for esophagogastric, *p value = 0.0284; ns, non-significant. (**B**) Breast cancer microarray raw data and clinical information from *GOBO* (see “Methods” section) were downloaded. Chin raw data was downloaded from *EMBL-EBI*. To assess distant metastasis-free survival (DMFS), all specimens were evenly divided into three groups to generate a Kaplan–Meier survival curve using in-house Python script. H2AX expression level was clustered based on the following distribution: low (H2AFX = 4.419–6.69, n = 296 samples; intermediate (H2AFX = 6.694–7.267, n = 296 samples, and high (H2AFX = 7.267 – 9.14, n = 295). (**C**) Expression level of *H2AFX* and *HK2* were extracted from the *GOBO* datasets described in (**B**) including samples without published medical history, and the analysis was performed using Spearman correlation coefficient. (**D**) Similar analysis was conducted using single-cell RNAseq dataset from *GSE176078* and specimens of triple-negative breast cancer (TNBC) were specifically considered for the analysis using Seurat library in R (n = cell number).

To investigate whether the role of H2AX in the regulation of HK2 expression and glycolysis may provide clues for any clinical correlation, we conducted H2AX expression level analysis in multiple cell lines derived from human malignancies using the *CCLE* dataset^30^. The data indicate that H2AX transcript is selectively overexpressed in cells derived from the metastatic sites of breast and esophagogastric cancers compared to the primary site, while H2AX level remains relatively unchanged in other cancer cells lines including cells derived from ovarian, pancreatic and colorectal cancers (Fig. 5A). These observations are substantiated by the evidence that high H2AX expression is associated with shorter distant metastasis-free survival in invasive breast cancers from the *GOBO* dataset (Fig. 5B). We also conducted an expression level comparison of H2AX and HK2 in invasive breast cancer specimens available in the *GOBO* dataset^21^, as well as in single cell-sequencing dataset of triple negative breast cancer patients (see GSE176078)^31^. Our data reveal that the transcript levels of H2AX and HK2 are positively correlated across all samples based on correlation coefficients (*GOBO*, r = 0.3410, p < 0.0001, n = 1434; GSE176078, r = 0.6372, p < 0.0001, n = 187, using the non-parametric Mann–Whitney test) (Fig. 5C,D). Collectively, these findings suggest a unique link between H2AX and HK2 in invasive breast cancers. Our data imply that H2AX loss leads to reduced cancer aggressiveness presumably via impairment of HK2-driven glycolytic metabolism and cancer cell’s ability to survive.

From the physiological standpoint, hexokinase-2 is an essential glycolysis gene regulated by multiple transcription factors including, but not limited to c-Myc, HIF1α, p63, and p53^11,32–35^. The repression of HK2 transcript observed upon H2AX loss implies a mechanism involving H2AX-mediated HK2 transcriptional control as revealed by repression of HK2 promoter activity upon H2AX loss (Supplementary Fig. 1). While requiring further study, these data raise an intriguing possibility that H2AX may either elicit a remodeling of the chromatin prior to enhanced transcriptional regulation of HK2; or H2AX phosphorylation may initiate a cascade of DNA damage response signaling involved in HK2 transcriptional control. Multiple lines of evidence have pointed to the notion that H2AX loss may result into an H2AZ exchange, prior to either activation of DNA damage responses, or to a transcriptional reprogramming required for the expression of several epithelial to mesenchymal transition genes, and metastasis^5,36–38^. While further investigations are needed, it should not be excluded a possibility of a profound change in the chromatin configuration which may involve other histone H2A variants exchange upon H2AX loss. Such chromatin remodeling may be part of events leading to HK2 transcriptional repression. It is also worth noting the existence of post-translational modifications involved in HK2 regulation. Examples include Akt-mediated HK2 phosphorylation^39,40^, or HectH9-mediated HK2 ubiquitination among many others^12^. While the present data tend to imply a transcriptional repression of HK2 upon H2AX loss, further investigation is needed to delineate mechanisms underlying HK2 regulation by the histone variant H2AX.

From the clinical standpoint, HK2 is a druggable target in multiple human malignancies. Several attempts have been made to develop small molecules inhibitors of HK2 for cancer therapy, including utilization of 2-Deoxy-d-glucose (2DG), but none of these agents have advanced beyond a phase II of clinical trials, presumably owing to their inability to induce irreversible inhibition of HK2^12,41^. Therefore, insights into genes or pathways coordinating HK2 regulation and its functions in glycolysis and resistance to treatment are necessary to foster development of new therapeutic strategies.

## Methods

### Cell culture and plasmid transient transfection

The human breast cancer cell line MDA-MB-231 (from ATCC, Manassas, VA USA), was grown at 37 °C with 5% CO_2_ in DMEM (Fisher Scientific, Waltham, USA), supplemented with 10% Fetal Bovine Serum (Thermo Scientific, Waltham, USA), while BT549 (from ATCC, Manassas, VA USA) was grown at 37 °C with 5% CO_2_ in RPMI-1640 (Cytiva, Marlborough, USA), supplemented with 10% Fetal Bovine Serum and 5 μg/ml insulin (Sigma-Aldrich, Burlington, USA). Cells were authenticated using *a colorimetric signal amplification system*, and tested for mycoplasma contamination (R&D systems, Minneapolis, USA). All media were supplemented with penicillin and streptomycin (Gibco, Amarillo, USA). For HK2 overexpression experiments, both MDA-MB-231 cells and BT549 H2AX cells were transfected using Lipofectamine^®^ 3000 (Thermo Scientific, Waltham, USA) following manufacturer’s instructions. pWZL-Neo-Myr-Flag-HK2 and pWZL-Neo-Myr-Flag-DEST vectors were purchased from Addgene (plasmid IDs: #20501, and #15300).

### Generation of stable H2A.X knocked-down cells

For lentiviral infection, HEK-293 FT cells were used for virus package according to the manufacturer’s instructions. To obtain H2AX-shRNA knockdown cells (shH2AX), or the control cell line (shCTRL), MDA-MB-231 or BT549 parental cells infected with pLKO.1-puro-H2AXshRNA (shH2AX); or control vector (shCTRL) were selected with puromycin (1 μg ml^−1^) for 1 week and the expression of H2AX was examined by western blotting. Briefly, the lentivirus packaging system was used as follows: lentiviral constructs that expressed shRNAs targeting H2AX; or scrambled shRNA were co-transfected with lentiviral packaging plasmids psPAX2 and pCMV-VSVG into HEK-293 FT cells using Lipofectamine^®^ 3000 (Thermo Scientific, Waltham, USA) according to the manufacturer’s instructions. At 48 h post-transfection, culture medium was collected to be incubated with MDA-MB-231 or BT549 cells in the presence of polybrene (8 μg ml^−1^). At 48 h post-infection, infected cells were either harvested for gene and protein expression analysis or selected with puromycin to establish stable clones. shH2AX: #TRCN0000073281 (Open Biosystems /Dharmacon, Thermo Scientific, Waltham, USA) was a kind gift of Dr. William M. Bonner, and has been previously tested for further validation.

### Generation of cells stably expressing luciferase for in vivo bioluminescence

For retroviral infection, HEK293 FT were used for virus packaging according to the manufacturer’s instructions. Briefly, the lentiviral constructs pFCAG-Luc hygro (Addgene #67502), pCMV-VSVG and psPAX2 were transfected into HEK-293 FT cells using Lipofectamine^®^ 3000 according to the manufacturer’s instructions; and viral particles were harvested at 48 h post-transfection. Cells were infected with virus for 48 h in the presence of polybrene (8 μg ml^−1^). Infected cells were selected to establish stable clones using hygromycin B (400 μg ml^−1^).

### Real-time PCR

Total RNA was extracted from cells using RNeasy Mini Kit (Qiagen, Valencia, USA) according to the manufacturer’s instructions. Quality of RNA preparation, based on the 28S/18S ribosomal RNAs ratio, was assessed using the NanoDrop one (Thermo Scientific, Waltham, USA). Reverse transcription was performed with High-Capacity cDNA Reverse Transcription Kit (Fisher Scientific, Waltham, USA). Real-time PCR (RT-PCR) was performed with TaqMan Gene Expression Master Mix (Thermo Scientific, Waltham, USA) according to the manufacturer’s instructions. Oligonucleotides were pre-designed, validated; and are considered to be proprietary information by Thermo Fisher Scientific. The assays IDs are available and are referenced as follow: ACTB (Hs01060665_g1), HK1(Hs00175976_m1), HK2 (Hs00606086_m1), H2AX (Hs00266783_s1).

### RNA-sequencing

Total RNAs from MDA-MB-231 or BT549 cells infected with scrambled shRNAs (shCTRL) or with shRNAs targeting H2AX (shH2AX) were extracted using RNeasy Mini Kit (Qiagen, Valencia, USA) following manufacturer’s instructions. All RNAs were QC-tested and library construction and sequencing were done by GSAF (Genomic Sequencing and Analysis Facility, the University of Texas at Austin).

### Sequencing data analysis

The raw sequencing data were evaluated by *FAST-QC* including quality distribution of nucleotides, position-specific sequencing quality, GC content, the proportion of PCR duplication, k-mer frequency, etc. Adaptor sequences and low-quality reads that contained more than 20% of bases with qualities of < 13 of the raw data were removed to obtain the clean reads. Clean reads were aligned to human genome (Version: UCSC hg19, RefSeq) with TopHat default parameters^42^. Aligned reads were analyzed with Cufflinks to get normalized transcript-level and gene-level expressions^43^. DESeq was applied to identify the DEGs according to FDR < 0.05 and log2 FC ≥|± 0.15|^44^. The commonly shared genes between DEGs and metabolism-related genes were conducted with in-house script, and biological interpretation and pathway analysis was performed with the *Ingenuity Pathway Analysis (IPA)* tool. Heatmap analysis for shared genes was applied using in-house script.

### Western blots

Cells were washed twice with PBS (Thermo Scientific, Waltham, USA), directly solubilized in denaturing sample buffer and then subjected to SDS-PAGE. Proteins were electro-transferred to 0.2 µm nitrocellulose sheets (Bio-Rad, Hercules, USA) for immunodetection with the following primary antibodies: H2A.X (1:5000; ab20669, Abcam, Cambridge, MA, USA); HK2 (1:1000; C64G5, Cell Signaling Technology Inc); Tubulin (1:2000; 11H10, Cell Signaling Technology Inc). Immune complexes were detected with goat coupled anti-rabbit or horse coupled anti-mouse IgG antibodies (Cell Signaling Technology Inc). Cropped images for the western blots and molecular weight are shown in the main and supplementary figures.

### Lactate measurement

50,000 cells were seeded in each well in 96-well plates. The medium was replaced 24 h post culture with either phenol red-free DMEM or phenol red-free RPMI-1640 (Invitrogen, Carlsbad, USA). All media were complemented with 10% dialyzed Fetal Bovine Serum (FBS, Atlanta Biologicals, GA, USA) for the experiment. Extracellular lactate production was measured 24 h following culture with phenol red-free medium using the Lactate Assay Kit (Eton Bioscience, San Diego, USA) acording to the manufacturer’s instructions.

### GSEA analysis

Normalized gene-expression levels from MBA-MB-231 and BT549 cells were performed separately by *GSEA4.1.0* using hallmark gene sets with default parameters. Consistency of gene sets with an FDR < 25% in both cell lines was considered.

### Apoptosis

10, 000 MDA-MB-231 cells were seeded in each well of 96-well tissue culture plate. Apoptosis level was measured by Caspase-Glo 3/7 assay (Promega, Wisconsin, USA) after 48 h based on the manufacturer’s protocol.

### Proliferation

5000 MDA-MB-231 cells were seeded in each well in of 96-well tissue culture plate. Proliferation level was measured 72 h post culture using *IncuCyte* (Sartorius, Gottingen, Germany) according to the manufacturer’s instruction.

### Promoter activity

Promoter activity of HK2 and ACTB was measured using *LightSwitch* promoter reporter GoClone system (SwitchGear Genomics, Carlsbad, USA) according to the manufacturer’s instruction. Positive control and negative control were provided by manufacturer.

### Model of experimental metastasis using MDA-MB-231-luciferase cells

All animals were treated in accordance with the recommendations of the National Institutes of Health and approved by the University of Texas at Austin Committee on Animal Care. All animal procedures were performed according to protocols approved by the University of Texas at Austin Animal Care and Use Committee (AICUC). Tail vein injection was performed as previously described^2^. Six to eight-week-old female immunocompromised NOD SCID gamma mice (Stock #: 005557, Jackson Laboratory, Bar Harbor, ME, USA) were injected with MDA-MB-231 CTRL cells expressing control vector (shCTRL), H2AX knockdown cells expressing control vector (shH2AX), and HK2 revertant cells (shH2AX + HK2 vector ) via the lateral tail vein using 29.5-gauge needles, and followed up for metastases burden. In brief, 1 × 10^6^ cells suspended in 200 µl DMEM were injected into the tail vein of each mouse. After 6 weeks, animals were sacrificed and examined macroscopically and microscopically for the presence of metastases. Tumor growth was monitored weekly using bioluminescence imaging. Briefly, mice were intraperitoneally (I.P.) injected with 100 mg/kg luciferin (in 200 μL) with 25G needle and imaged using the IVIS Lumina instrument in the procedure room. Prior to the imaging, mice are anesthetized by using isoflurane for approximately less than 15 min at a time.

All mice were sacrificed using a CO_2_ chamber and tissues were collected. Upon collection, tissues were fixed in 10% normal buffered formalin followed by sections and H&E immunostaining. During the procedures, any animal experiencing rapid weight loss (greater than 20%, measured weekly), debilitating diarrhea, rough coat, hunched posture, labored breathing, lethargy, persistent recumbence, jaundice, anemia, significantly abnormal neurological signs, bleeding from any orifice, self-induced trauma, impaired mobility, or has difficulty obtaining food or water was immediately euthanized. In addition, mice were euthanized when they became moribund.

No human participant was involved in this study. All methods are reported in accordance with ARRIVE guidelines for the reporting of animal experiments.

### Database analysis

Breast cancer microarray raw data and clinical information from *GOBO* including *GSE1121, GSE2034, GSE2603, GSE5327, GSE6532, GSE7390 and GSE12093* were downloaded from *GEO*. Chin raw data (E-TABM-158) was downloaded from *EMBL-EBI*. Probes signals were extracted with Expression Console Software (Affymetrix, Thermo Fisher) and batch effects were removed by Limma library in R. Expression level of H2AFX, HK2, HK1 were extracted from these datasets and analyses were performed to using spearman correlation analysis. To assess distant metastasis-free survival DMFS (all specimens were divided evenly into three groups based on H2AX expression level to generate a Kaplan–Meier survival curve using in-house Python script. Cancer Cell line Encyclopedia (CCLE) normalized expression data and clinical information from MIT-Broad Institute were downloaded from cBioportal. Single-cell dataset *GSE176078* was downloaded from GEO and specimens of triple-negative breast cancer (TNBC) were considered for the analysis using Seurat library in R^45^, and cells concurrently expressing H2AX, HK2 and ACTB were used for the analysis.

### Immunohistochemical analysis

Sections were deparaffinized and rehydrated, and antigen retrieval was performed with citrate buffer in a water bath at 120 °C. The sections were incubated with the anti-KI67 antibody (for 1:200, Cell signaling) overnight at 4 °C and then incubated with a biotinylated secondary antibody for one hour at room temperature (Vectastain Elite Rabbit, Vector Laboratories, Burlingame, CA). The slides were developed with diaminobenzidine (Vector Laboratories, Burlingame, CA), and counterstained with hematoxylin. The slides were scanned at 20× magnification.

### Statistical analysis

Statistical analyses were performed using GraphPad Prism 6 software (GraphPad Software). A value of p < 0.05 was considered statistically significant. Data are presented as mean ± S.E.M.

### Ethical approval

All methods were carried out in accordance with relevant guidelines and regulations.

## Supplementary Information

Supplementary Figures.
