# Supplementary material for: Histone H2AX promotes metastatic progression by preserving glycolysis via hexokinase-2
Source: Sci Rep. 2022 Mar 8;12:3758. doi: 10.1038/s41598-022-07675-6 (PMC8904825; doi:10.1038/s41598-022-07675-6)

**Supplementary information**

**Histone H2AX promotes metastatic progression by preserving glycolysis via hexokinase-2**

Yue Liu^1,2^, Haojian Li^1,2^, Crystal N. Wilson^1,2^, Hui Jen Bai^1,2^, Myriem Boufraqech^1,2^, and Urbain Weyemi^1,2*^

1, Department of Molecular Biosciences, The University of Texas at Austin, Austin, Texas 78712, USA. 2, Institute for Cellular and Molecular Biology, The University of Texas at Austin, Austin, Texas 78712, USA.

* Corresponding author:

Urbain Weyemi, Ph.D.

Department of Molecular Biosciences

University of Texas at Austin

2506 Speedway

Austin, TX 78712

Phone: 512-232-7706

Email : urbain.weyemi@austin.utexas.edu

**
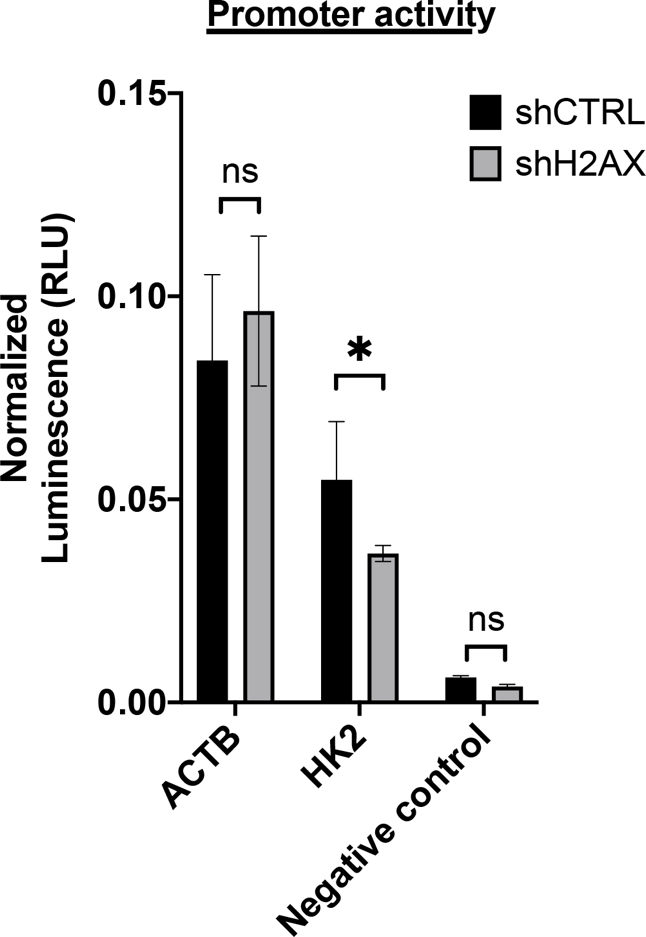
**

**Supplementary Figure 1. H2AX depletion leads to impaired Hexokinase-2 (HK2) promoter activity.** H2AX depletion reduces the promoter activity of HK2, but not ACTIN. HK2 and ACTIN promoter activities were accessed by luciferase reporter assay in control cells (shCTRL) and in cells silenced for H2AX (shH2AX). Error bars represent the s.e.m. (*n*=3). Statistical significance was determined by two-stage unpaired Student’s *t*-test.

**
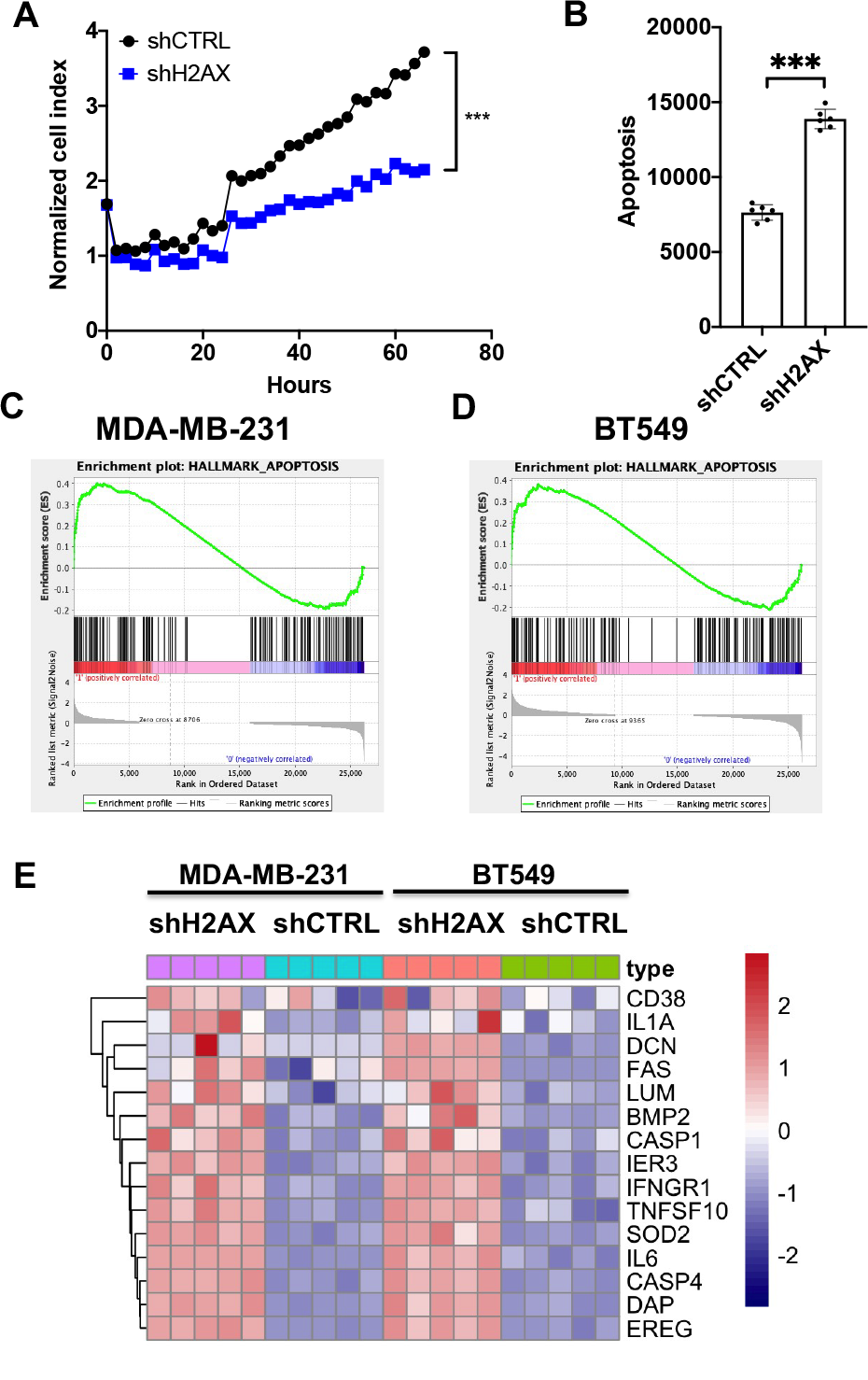
**

**Supplementary Figure 2. H2AX depletion leads to reduced proliferation and enhanced apoptosis in triple-negative breast cancer cells.** A. MDA-MB-231 cells (shCTRL and shH2AX) were seeded in each well in of 96-well tissue culture plate. Proliferation level was measured 72 hours post culture using *IncuCyte* . Error bars represent the s.e.m. (*n*=3). Statistical significance was determined by a two-tailed, unpaired Student’s *t*-test. B. MDA-MB-231 cells (shCTRL and shH2AX) were seeded in each well of 96-well tissue culture plate. Apoptosis level was measured by Caspase-Glo 3/7 assay. C, D. Gene set enrichment analysis (GSEA) was performed by using H2AX depletion-specific signature showing significant enrichment of apoptosis gene set after H2AX loss. E. Heatmap of apoptosis genes differentially expressed between control and H2AX-deficient cells.

**
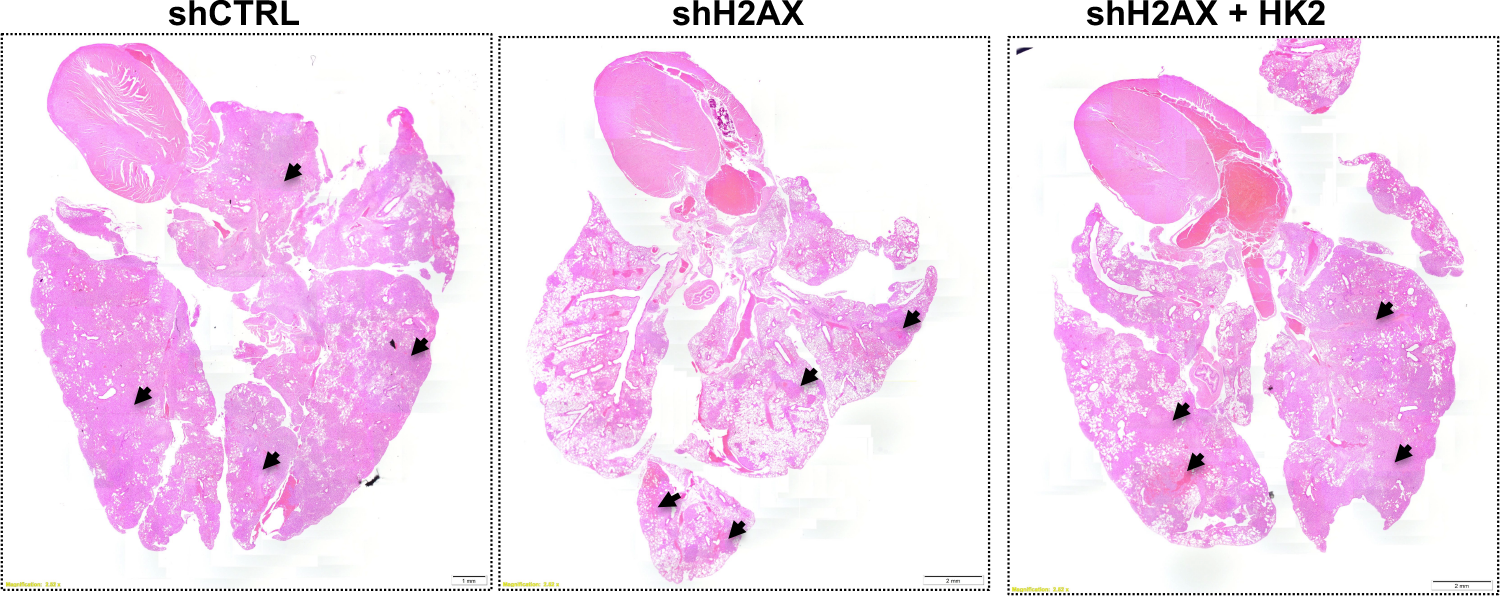
**

**Supplementary Figure 3.** Representative H&E-stained lung sections from mice injected with control cells with empty vector (shCTRL), H2AX-deficient cells with empty vector (shH2AX) and H2AX-deficient cells in which HK2 was re-expressed (shH2AX + HK2). Scale bars, 2 mm. Arrows indicate microscopic lung nodules.

**Supplementary Figure 4: Uncropped scans of western blot results displayed in this manuscript**

**
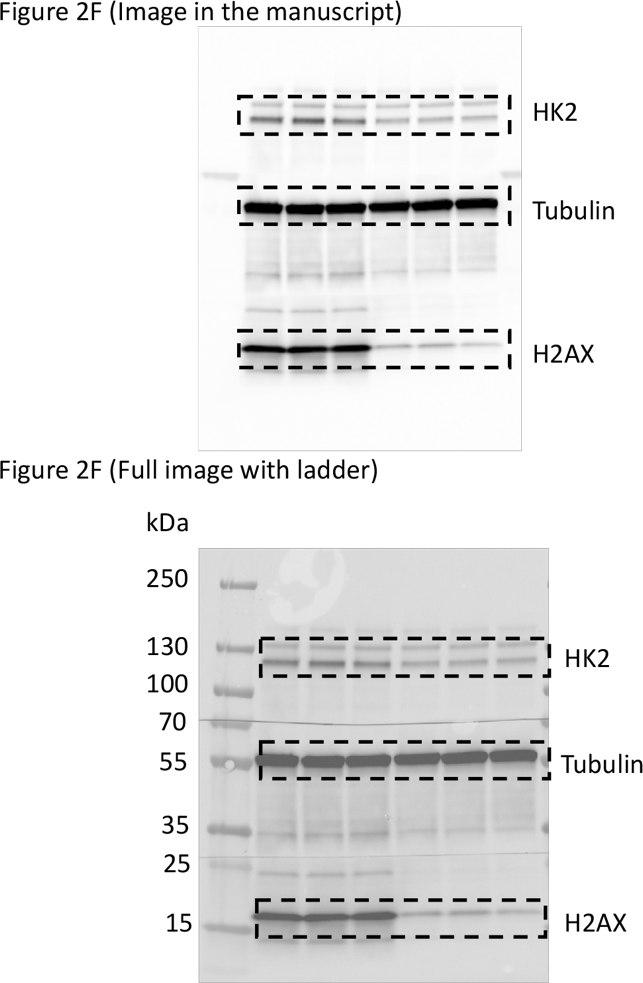
**


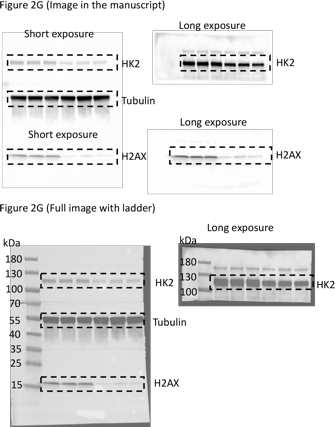


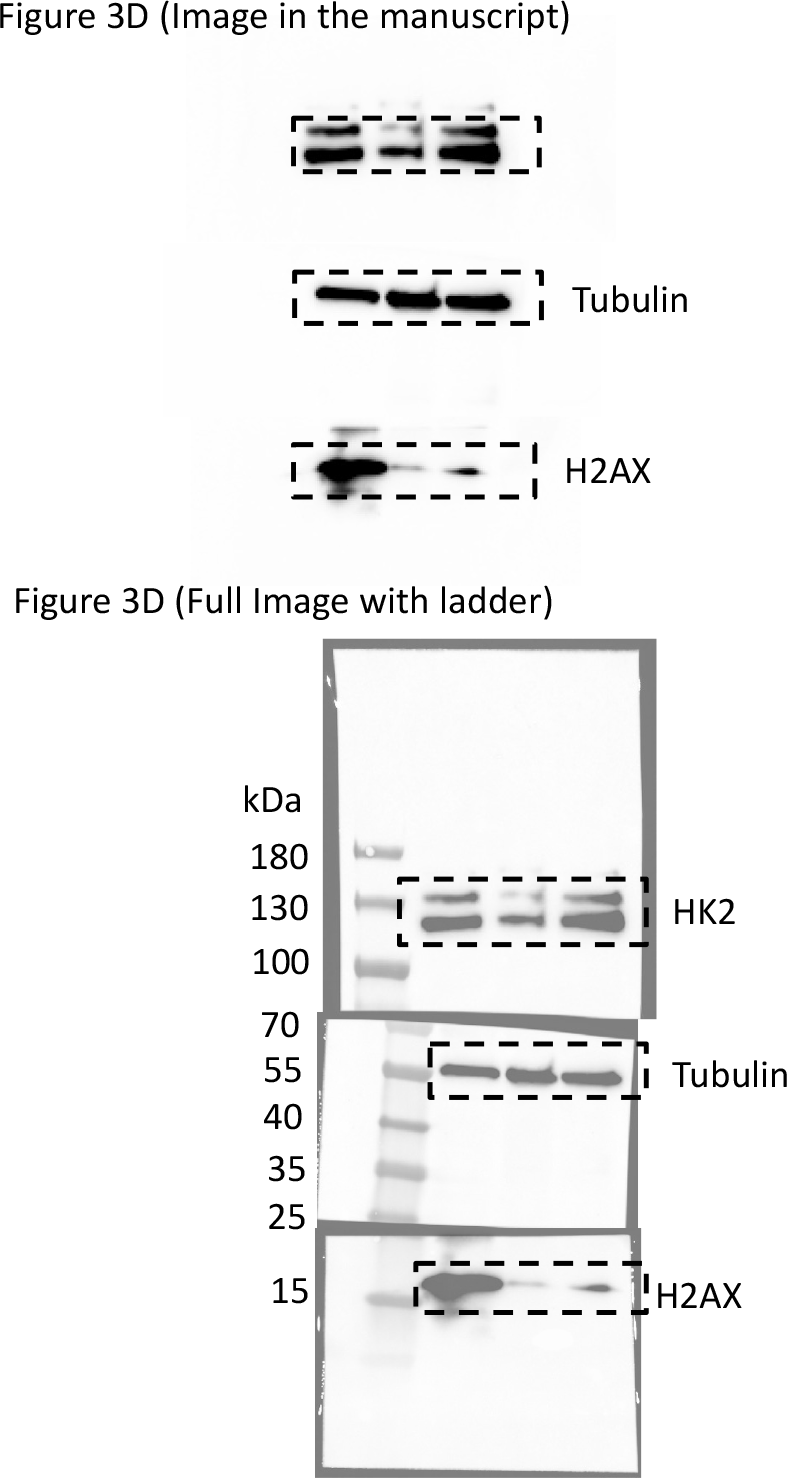

Supplement: Supplementary file 1 — Supplementary Figures. [file 41598_2022_7675_MOESM1_ESM.doc]
